# Declining malaria in Africa: improving the measurement of progress

**DOI:** 10.1186/1475-2875-13-39

**Published:** 2014-01-30

**Authors:** Peter W Gething, Katherine E Battle, Samir Bhatt, David L Smith, Thomas P Eisele, Richard E Cibulskis, Simon I Hay

**Affiliations:** 1 Department of Zoology, Spatial Ecology and Epidemiology Group, Tinbergen Building, University of Oxford, South Parks Road, Oxford, UK; 2 Malaria Research Institute, Johns Hopkins Bloomberg School of Public Health, Baltimore, MD, USA; 3 Fogarty International Center, National Institutes of Health, Bethesda, MD, USA; 4 Department of Global Health Systems and Development, Center for Applied Malaria Research and Evaluation, Tulane University School of Public Health and Tropical Medicine, New Orleans, LA, USA; 5 Global Malaria Programme, World Health Organization, Geneva, Switzerland

## Abstract

The dramatic escalation of malaria control activities in Africa since the year 2000 has increased the importance of accurate measurements of impact on malaria epidemiology and burden. This study presents a systematic review of the emerging published evidence base on trends in malaria risk in Africa and argues that more systematic, timely, and empirically-based approaches are urgently needed to track the rapidly evolving landscape of transmission.

## Background

The last decade has witnessed a dramatic rise in commitment to malaria control in Africa, with financing increasing approximately twentyfold since the year 2000 [1,2]. Although these funding levels remain inadequate [2,3], they have over this period enabled the distribution of more than half a billion insecticide treated mosquito nets, financed insecticide spraying campaigns in nearly all endemic African countries, improved access to curative and preventative anti-malarial drugs for millions of people at risk, and contributed to broader health system strengthening efforts [2]. This escalation of control activities has increased the importance of accurate measurements of impact on malaria epidemiology and burden. This study presents a systematic review of the emerging published evidence base on trends in malaria risk in Africa and argues that more systematic, timely, and empirically-based approaches are urgently needed to track the rapidly evolving landscape of transmission. In particular, this review presents two central arguments: (i) that empirical studies measuring change are biased towards low transmission settings and not necessarily representative of high-endemic Africa where declines will be hardest-won; and (ii) that current modelled estimates of broad scale intervention impact are inadequate and now need to be augmented by detailed measurements of change across the diversity of African transmission settings. In line with the notion that improving the measurement of progress is vital in sustaining that progress [4], this piece highlights emerging opportunities to strengthen and utilize more systematically both survey and routine surveillance measurement systems to better enumerate changing patterns of malaria risk across the continent [3,5-7].

### Evaluating the published empirical evidence base

A variety of malaria-related data have been used to assess change. A natural starting point is to examine trends in routine surveillance data on cases and deaths recorded by health systems, but such data are not yet considered sufficiently reliable to track change in nearly all endemic countries in Africa [2]. One alternative has been to bring together the many *ad-hoc* measurements of change that have been generated over the past decade in Africa by researchers, national programmes and other agencies. Such studies range in scale from single villages, to large multi-centre trials, to national-level analyses of health system data, and report on trends in transmission intensity, infection prevalence and malaria morbidity and mortality. The first major attempt to assemble and summarize these observations, by O’Meara and colleagues [8], found that a majority reported declines of some sort, and this work has subsequently become one of the most widely cited research outputs on the changing epidemiology of malaria in Africa. However, this assembly of studies was opportunistic: the geographical coverage of observations necessarily reflected the locations where research has been concentrated. This non-random coverage is important because, taken together, these data are potentially biased and may not capture the potentially complex patterns of change across the continent.

To evaluate the extent that available research data are representative of endemic Africa as a whole, a systematic review was conducted of all primary research studies assessing trends in malaria transmission or burden. This was designed to replicate the search and inclusion criteria of O’Meara *et al*. [8], but also to update the assembly to include studies published up to March 1 2013. A full description of the methodology and results is included in Additional file 1, with a spreadsheet of studies in Additional file 2. In brief, broad search terms were used on metrics of malaria transmission or burden to identify 641 references which were screened and subject to successive exclusion criteria to yield a total of 89 published studies (44 added to the 45 of O’Meara *et al.*) reporting primary measurements of change in sub-Saharan Africa since the year 2000. The geographical area represented by each measurement was then identified and digital boundaries created for those areas within a geographical information system. To assess whether those combined areas were representative of transmission settings across the wider continent, they were then overlaid on an endemicity map [9] and a comparison made between the statistical distribution of endemicity (*Plasmodium falciparum* parasite rate in 2–10 year olds, *Pf*PR_2-10_) within study areas assessing change versus that for endemic Africa as a whole.

**Figure 1:**
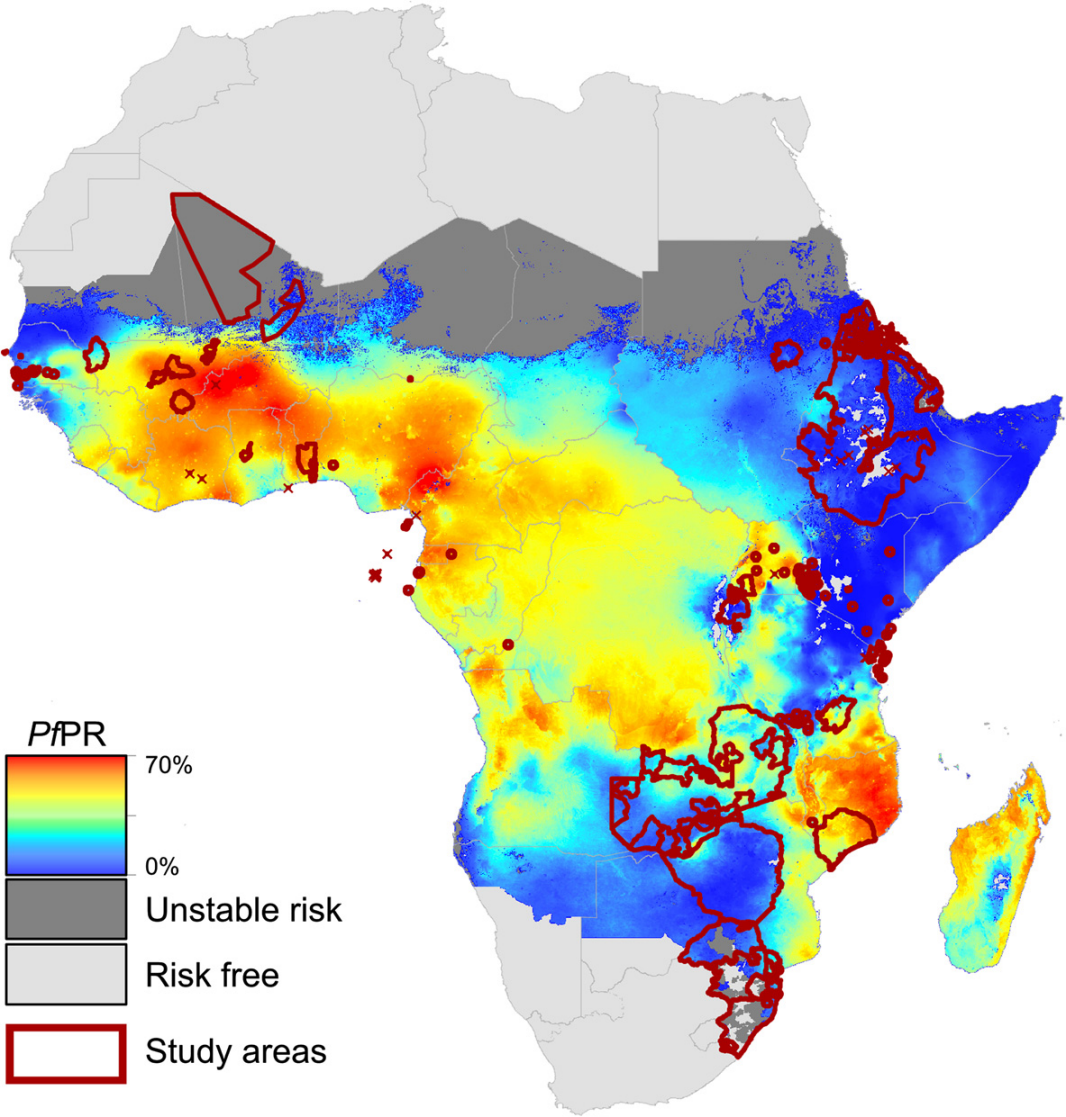
**Geographical distribution of studies measuring changing malaria risk in Africa, shown overlaying a predicted surface of endemicity (*P. falciparum *** **parasite rate, *Pf*** **PR)**[9]**.**

**Figure 2:**
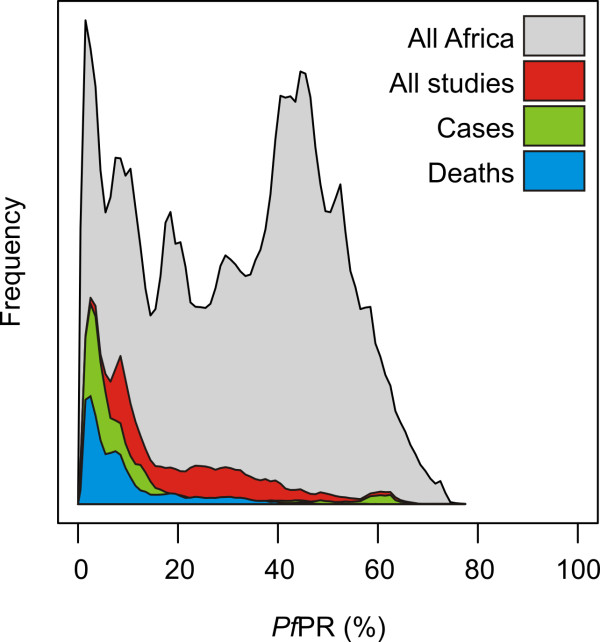
**Histograms comparing the statistical distribution of predicted *P. falciparum *parasite rate (*Pf*PR) across endemic Africa as a whole (grey), and within study sites representing measurements of changing malaria risk via any metric (red), cases (green), and deaths (blue).** Frequency is measured by number of 5 × 5 km pixels.

The combined study area represented by measurements of change was 3.6 million km^2^ (Figure 1), approximately 16% of the area of Africa at any risk of malaria [9]. The level of endemicity within these studied areas (mean *Pf*PR_2-10_ = 16%) was systematically lower than across the continent as a whole (mean *Pf*PR_2-10_ = 31%) (Figure 2). While 40% of endemic Africa experienced ‘high-endemic’ transmission in 2010 (*Pf*PR_2-10_ in excess of 40%) [9], only 9% of the studied areas were from these high transmission settings. This tendency was more pronounced for studies reporting trends in malaria morbidity or mortality. Only 6% and 2% of areas studied for trends in cases and deaths, respectively, were in high endemic settings. Conversely, 46% (cases) and 43% (deaths) of those areas were in low-endemic settings (*Pf*PR_2-10_ below 5%) which comprise only 11% of the area at risk across Africa [9]. This over-representation of low-endemic settings means that caution should be exercised when seeking to summarize or extrapolate the published empirical evidence base on changing malaria across the continent. Some 327 million Africans (41% of the total population at risk) live in areas that remain high-endemic [9], and current understanding of how malaria may be changing in these settings is based on limited published evidence, particularly for cases and deaths.

### Evaluating the modelled evidence base

Perhaps the most informative attempts to explore more systematically how malaria burdens in Africa may be changing across different transmission settings have come not from direct measurements, but from a variety of modelling efforts. One set of approaches has combined data on intervention coverage with estimates of the effectiveness of those interventions in reducing morbidity or mortality, derived primarily from controlled trials. For the high-endemic African countries with weak surveillance data, for example, the World Health Organization (WHO) configures a baseline estimate of case incidence for the year 2000 [10]. Estimates are then progressively downgraded in each subsequent year according to growing intervention coverage levels within each country, assuming effects match those seen in controlled trials [2]. A conceptually similar approach has been taken using the Lives Saved Tool [11,12] to estimate the contribution of trends in malaria-attributable deaths to observed declines in all-cause child mortality. These tools deliver on their intended purpose: to provide a broad-scale picture of the plausible impact of malaria control scale-up on cases and deaths. As such, they have addressed an information shortfall needed to support international policy setting and advocacy.

However, an important message in these studies is sometimes overlooked when interpreted by a wider audience: the vital distinction between analyses predicated on assumed impact and empirical evidence for that impact. Studies of this type employ the pragmatic assumption that estimates of intervention protective efficacy derived from controlled trials [13] or pooled household survey data [14] can be applied continent-wide to predict resultant impacts on burden through time. The use of these averaged effect sizes means that this approach is well suited to assessing trends at broad scales. At finer scales, assessing intervention impact by assuming a given level of impact *a priori* means it may not be possible to identify settings where control measures perform better or worse than expected. Importantly, this limits iterative refinement and optimization of strategies in response to local scale heterogeneities in both baseline risk and subsequent response to intervention efforts.

Other modelling studies have used data on causes of death to estimate how the contribution of malaria to all-cause mortality has changed through time [15]. For African countries, such data are rare and analyses rely on a limited number of post-mortem verbal autopsy studies that are known to have limitations and sources of misclassification bias [16]. These studies are important contributions to the evaluation of the relative contributions of malaria mortality against other leading causes of death. Again, however, the inherent limitations of the input data mean analysis of change is inevitably broad scale and contingent on assumptions that are hard to verify across diverse local settings until more robust and geographically detailed data on cause-specific mortality become available.

## Discussion

Despite the inherent difficulties in measuring changes in malaria in Africa, several aspects are not in doubt. It is clear that that an immense international effort has yielded dramatic increases in the coverage of malaria control interventions, and that these interventions are effective at reducing malaria transmission, case incidence, and death. However, it is also clear that that their coverage remains variable and considerably below stated targets [2,3], and that the efficacy of a given intervention strategy will vary according to local entomological, epidemiological, and health system factors. What is the effect of these interacting factors on patterns of change across the continent? This crucial question has only been answered to a limited extent using data and methods with important caveats. Scrutiny of the existing evidence indicates that directly observed data on declining malaria are most abundant in lower transmission settings where progress is likely to be more straightforward, and measurement and analysis need to become more representative of the spectrum of African endemicities. Similarly, modelled predictions of impact need further validation and refinement against a contemporary and geographically detailed empirical evidence base.

A more complete understanding of the changing pattern of transmission is becoming progressively more important for several reasons. As funding comes under pressure, donor agencies must demonstrate that investments are yielding impact, and that this impact can be tracked robustly [17-19]. National programs looking to stratify their approaches to malaria control [3] require detailed monitoring of changing patterns of risk to plan adaptation as local conditions evolve. Reliance on broad assessments means that local heterogeneities may be overlooked, potentially encouraging a one-size-fits-all response when tailored control efforts may be more effective [20]. The importance of understanding and responding to shifting heterogeneities in risk has been underscored by the Roll Back Malaria Global Malaria Action Plan which calls for stratified, targeted, and locally appropriate strategies for control [3]. In parallel, the establishment by the WHO Malaria Policy Advisory Committee of an Evidence Review Group on Malaria Burden Estimation [21] has provided renewed focus on the challenges and opportunities of tracking change in Africa.

This increasing recognition of the importance of robust routine data [5] means national surveillance systems in Africa can continue to strengthen, aided by improving healthcare access, diagnostic capacity, and rapidly evolving communication technologies [7]. Meanwhile, investments in major survey programs have allowed many African countries to undertake multiple rounds of cross-sectional household surveys, increasingly with both cluster-level GPS coordinates and malaria blood testing [22]. These surveys have not been included in our review because they represent raw data rather than analysis of change. However, their increasing availability provides an unprecedented opportunity to conduct spatiotemporally detailed evaluations of changing infection prevalence co-measured with intervention coverage. Such data, augmented by ongoing theoretical and observational analyses of how interventions interact with the cycle of transmission and resultant human disease, have the potential to transform understanding of how malaria in Africa is changing. These opportunities must be realized, sustained, and converted into effective decision-making. This will rely on not only a renewed international commitment to financial and programmatic support for surveillance and survey activities in Africa but a drive by the scientific community to develop systematic and geographically consistent approaches to analysing the resulting data, rather than relying on ad-hoc amalgams of local studies. Modelling has an important role, but the communication of assumptions and uncertainties in engagement with decision makers must be improved.

The coming years will see threats to progress – financial, climatic, evolutionary – line up against technological and political opportunities to consolidate and extend our successes. It has never been more important to develop accurate, timely, and representative mechanisms for measuring change capable of addressing these challenges.

## Competing interests

The authors declare that they have no competing interests.

## Authors’ contributions

PWG and SIH conceived the study. PWG and KB conducted the systematic review and undertook the data summary analysis. SB, DLS, TPE and REC contributed ideas and interpretation. PWG wrote the first draft of the manuscript and all authors contributed to its refinement and approved the final version.

## Supplementary Material

Additional file 1 **This Word document includes additional details of the materials and methods used in the study, along with additional results and figures. Figure S1A.** Schematic overview of the literature review procedure and results to assess changes in malaria transmission in sub-Saharan Africa as of 2013. **Figure S2A.** The distribution of study sites included in the review of changes in malaria transmission in sub-Saharan Africa in 2013. Study areas that assessed change in malaria transmission at the country level are shown in white. Regions that were digitized from a combination of administrative units or from paper maps are shown in the lightest green. Level 1-3 administrative units are shown in as polygons in graduated shades of green. Health facility studies have been shown as 25 km^2^ catchment areas, shown as green circles and cross-sectional surveys conducted at point locations are displayed as dark green triangles. Click here for file

Additional file 2 **This Excel spreadsheet tabulates in full the results of our systematic literature review on studies reporting measured changes in metrics of *P. falciparum *** **in Africa.** Click here for file
