# Supplementary material for: Declining malaria in Africa: improving the measurement of progress
Source: Malar J. 2014 Jan 30;13:39. doi: 10.1186/1475-2875-13-39 (PMC3930350; doi:10.1186/1475-2875-13-39)
Supplement: Additional file 1 — This Word document includes additional details of the materials and methods used in the study, along with additional results and figures. Figure S1A. Schematic overview of the literature review procedure and results to assess changes in malaria transmission in sub-Saharan Africa as of 2013. Figure S2A. The distribution of study sites included in the review of changes in malaria transmission in sub-Saharan Africa in 2013. Study areas that assessed change in malaria transmission at the country level are shown in white. Regions that were digitized from a combination of administrative units or from paper maps are shown in the lightest green. Level 1-3 administrative units are shown in as polygons in graduated shades of green. Health facility studies have been shown as 25 km2 catchment areas, shown as green circles and cross-sectional surveys conducted at point locations are displayed as dark green triangles. [file 1475-2875-13-39-S1.doc]

Additional Material for:

# Declining malaria in Africa: improving the measurement of progress

Peter W. Gething, Katherine E. Battle, Samir Bhatt, David L. Smith, Thomas P. Eisele, Richard E. Cibulskis, Simon I. Hay

**This file includes:**

- Additional Materials and Methods

- Additional Results

- Additional Figures A1, A2

- References for Database A3 (Additional File 2)

**Materials and Methods**

**Literature search**

A literature search was performed to update the findings of the review performed by O’Meara, *et al.*[8]. O’Meara and colleagues evaluated studies reporting changes in malaria endemicity through prevalence, incidence and slide positivity rate (SPR) measures. The original 46 studies used by O’Meara, *et al.* were identified and included in this review.

The updated literature search was conducted in PubMed on 12 December 2012 and repeated on 1 March 2013. The criteria used were based on those outlined by O’Meara and colleagues. The following Medical Subject Heading (MeSH) terms were included: “Africa South of the Sahara” and “malaria/epidemiology” or “malaria/mortality” or “malaria/prevention and control” or “malaria/transmission” not “malaria/immunology”, limiting to studies published after 1 January 2000. To not repeat the efforts of O’Meara, *et al*., all references published before 2010 were removed. The titles of the remaining references were reviewed for relevancy. Articles selected for inclusion were those that reported at least two years of data on malaria-specific indicators: clinical or slide diagnosed case numbers, entomological inoculation rate (EIR), incidence, prevalence through *Plasmodium falciparum* parasite rate (*Pf*PR), malaria specific mortality or seroconversion rate (SCR) or SPR. Studies that were excluded were cross-sectional surveys reporting a single time point, intervention studies covering less one year of follow up, entomological studies without any clinical or parasitological components or with only a single EIR and studies published since 1999 that did not report data from that time period. Note that these inclusion and exclusion criteria have been modified slightly from those used by O’Meara and colleagues, particularly in the inclusion of entomological data and data from pregnant women. Following the check of titles, abstracts were assessed and, from those, the full-texts to be included in the review were identified. These were combined with the 46 references from O’Meara, *et al*. However, one reference that was an abstract only was excluded due to insufficient data. A summary of the literature search process is summarised in Figure A1.

**Data abstraction**

Data abstracted from each study included information regarding the location, data source (e.g. community versus health facility), study period, population size, age range, reported endemicity, control measures applied, independent factors that may have affected transmission (including climatic data where provided), reported drug or insecticide resistance, the type of malaria metric used, whether cases were slide confirmed and the percent change described.

**Geopositioning and GIS extractions**

The area of each study location was plotted in a geographic information system (GIS). Point locations were used for community surveys carried out in named towns or villages. The latitude and longitude of the sites were obtained through Google Maps^TM^ (maps.google.com). Where studies were facility-based, we defined the representative study area as a 25 km^2^ buffer around each facility. Studies performed at a regional or national level were plotted as polygons by identifying Global Administrative Unit Layers (GAUL) codes implemented by the Food and Agriculture Organization of the United Nations. Data that originated from areas that could not be identified as an administrative unit were digitized using maps provided in the original documents or by combining existing administrative units. Figure A2 illustrates the locations of the various study sites across Africa.

Measures of *P. falciparum* transmission intensity for each study area were extracted from a predicted surface of *P. falciparum* parasite rate in 2 to 10 year olds (*Pf*PR_2-10_) in 2010 [9]. The combined study site locations were aggregated into a single ArcGIS (ESRI 2010) shapefile that was then converted into a raster grid. The site grid was overlayed on the prevalence surface so that an estimate of *P. falciparum* intensity was obtained for each 5 × 5 kilometre pixel of the study areas, from which summary histograms were computed.

**Additional Results**

**Literature review**

There were 641 references returned from the literature search that were published since 2010. Of the 119 titles selected for abstract review, 41 were excluded because there was only one year or less of follow up, 12 did not measure change over time and 11 did not measure malaria transmission data. Fifty-five full texts were evaluated and from those 44 references were included in the analysis. From the 55 full texts, two were excluded from less than one year of follow up, six did not measure change, two did not measure malaria transmission data and one reference was already included in the review by O’Meara, *et al.*.

**Data abstraction**

Data were extracted from 89 full texts, 44 obtained from the literature search and 45 from the review by O’Meara, *et al*. Because some references included data from more than one unique location, a total of 383 records of change in malaria transmission were obtained (listed in External Database S2). Eight different measures of malaria transmission were included. Five records used EIR as a measure of change, 145 used incidence, 54 malaria-specific deaths, two mean case values, 89 numbers of cases, 56 *Pf*PR, four SCR and 28 SPR. Of the records reporting on incidence and number of cases, 34% and 62% were slide confirmed, respectively. The majority (n = 271) of the 383 records were obtained from retrospective studies. The second largest majority were cross-sectional surveys (n = 88).

**Geopositioning and GIS extractions**

The majority of the study records were digitized from administrative units. Seven were done at the country level: two in Eritrea, two Rwanda, and one each for Djibouti, Zambia and Zimbabwe. Data in 23 records were collected at the first administrative unit (ADMIN 1) level, 99 at ADMIN 2 and four at ADMIN level 3. Study locations for 45 records were digitized and 137 were plotted as 25 km^2^ hospital catchment areas. The remaining 68 records were mapped as point locations. A total of approximately 3.6 million km^2^ of land is covered by the study areas.

**Figure A1** Schematic overview of the literature review procedure and results to assess changes in malaria transmission in sub-Saharan Africa as of 2013.


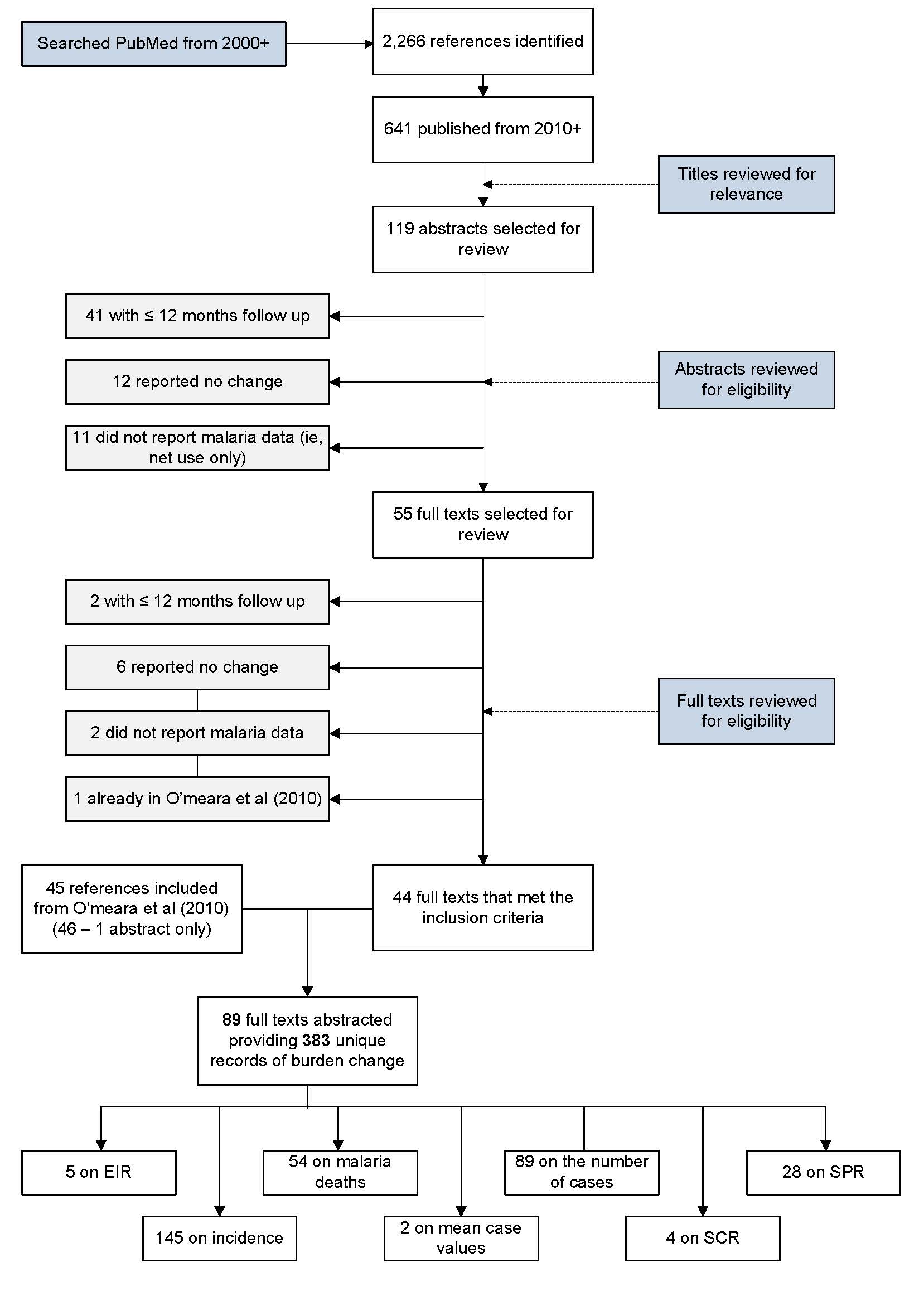


**Figure A2.** The distribution of study sites included in the review of changes in malaria transmission in sub-Saharan Africa in 2013. Study areas that assessed change in malaria transmission at the country level are shown in white. Regions that were digitized from a combination of administrative units or from paper maps are shown in the lightest green. Level 1-3 administrative units are shown in as polygons in graduated shades of green. Health facility studies have been shown as 25 km^2^ catchment areas, shown as green circles and cross-sectional surveys conducted at point locations are displayed as dark green triangles.


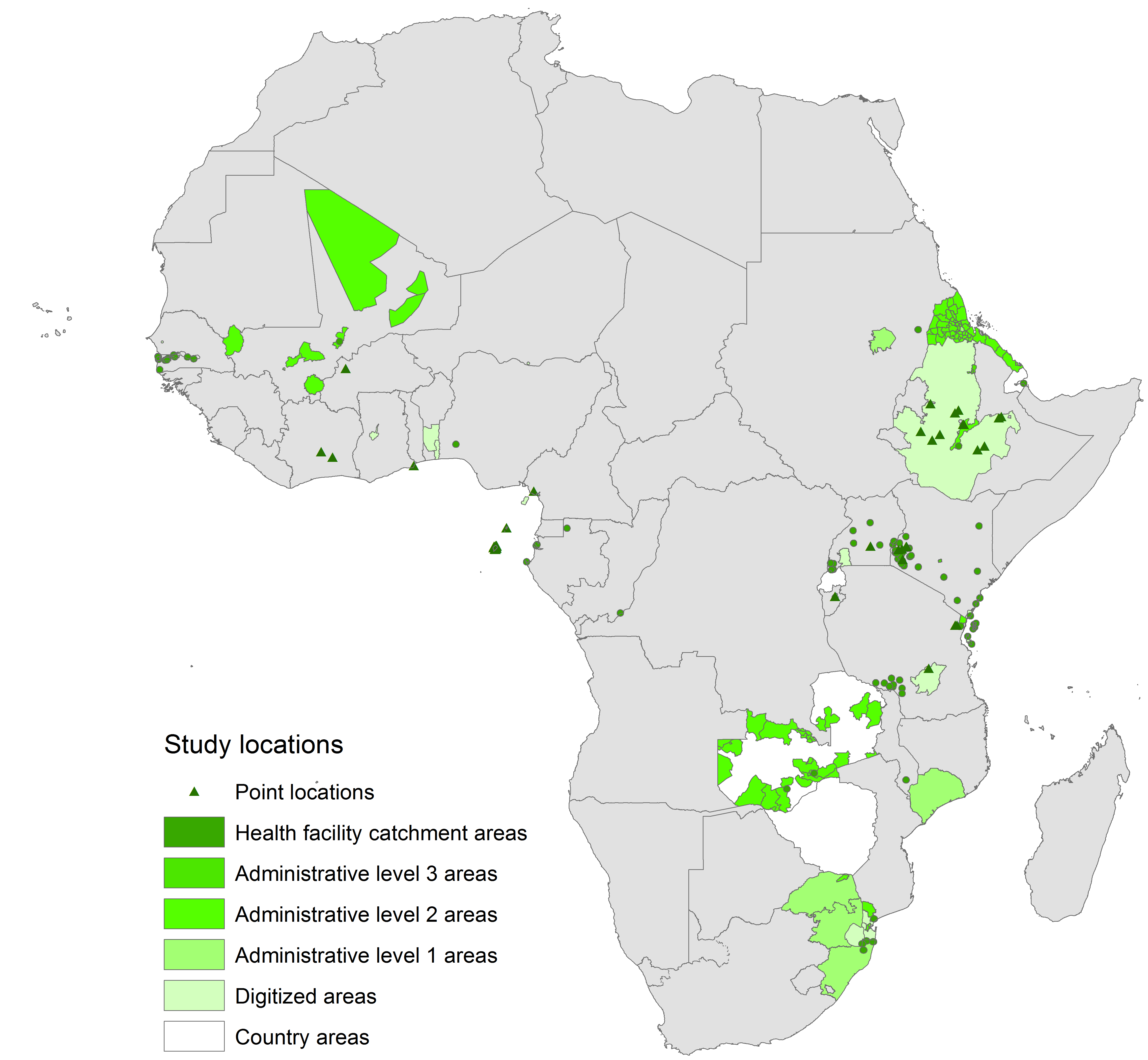


**Additional references for External Databse A3 (Additional File 2)**

The following studies are cited in the accompanying spreadsheet.

1. K. Adazu *et al.*, paper presented at the American Society of Tropical Medicine and Hygiene, 57th Annual Meeting, New Orleans, Louisiana, USA, Dec 7–11, 2008 2008.

2. C. K. Ahorlu, K. A. Koram, A. Seake-Kwawu, M. G. Weiss, Two-year evaluation of Intermittent Preventive Treatment for Children (IPTc) combined with timely home treatment for malaria control in Ghana. *Malar. J.* **10**, 127 (2011).

3. S. Alba, M. W. Hetzel, R. Nathan, M. Alexander, C. Lengeler, Assessing the impact of malaria interventions on morbidity through a community-based surveillance system. *Int. J. Epidemiol.* **40**, 405 (2011).

4. A. Alemu, G. Abebe, W. Tsegaye, L. Golassa, Climatic variables and malaria transmission dynamics in Jimma town, South West Ethiopia. *Parasit. Vectors.* **4**, 30 (2011).

5. M. S. Alilio *et al.*, Malaria control at the district level in Africa: the case of the muheza district in northeastern Tanzania. *Am. J. Trop. Med. Hyg.* **71**, 205 (2004).

6. D. Alonso, M. J. Bouma, M. Pascual, Epidemic malaria and warmer temperatures in recent decades in an East African highland. *Proc. R. Soc. Biol. Sci. Ser. B* **278**, 1661 (2011).

7. N. Amek *et al.*, Spatial and temporal dynamics of malaria transmission in rural Western Kenya. *Parasit. Vectors.* **5**, 86 (2012).

8. M. W. Aregawi *et al.*, Reductions in malaria and anaemia case and death burden at hospitals following scale-up of malaria control in Zanzibar, 1999-2008. *Malar. J.* **10**, 46 (2011).

9. K. I. Barnes *et al.*, Effect of artemether-lumefantrine policy and improved vector control on malaria burden in KwaZulu-Natal, South Africa. *PLoS Med.* **2**, e330 (2005).

10. Q. Bassat *et al.*, Malaria in rural Mozambique. Part II: children admitted to hospital. *Malar. J.* **7**, 37 (2008).

11. P. Bejon *et al.*, Stable and unstable malaria hotspots in longitudinal cohort studies in Kenya. *PLoS Med.* **7**, e1000304 (2010).

12. A. Bhattarai *et al.*, Impact of artemisinin-based combination therapy and insecticide-treated nets on malaria burden in Zanzibar. *PLoS Med.* **4**, e309 (2007).

13. M. K. Bouyou-Akotet *et al.*, Evidence of decline of malaria in the general hospital of Libreville, Gabon from 2000 to 2008. *Malar. J.* **8**, 300 (2009).

14. P. Brasseur *et al.*, Changing patterns of malaria during 1996-2010 in an area of moderate transmission in southern Senegal. *Malar. J.* **10**, 203 (2011).

15. H. Bukirwa *et al.*, Assessing the impact of indoor residual spraying on malaria morbidity using a sentinel site surveillance system in Western Uganda. *Am. J. Trop. Med. Hyg.* **81**, 611 (2009).

16. S. J. Ceesay *et al.*, Changes in malaria indices between 1999 and 2007 in The Gambia: a retrospective analysis. *Lancet* **372**, 1545 (2008).

17. S. J. Ceesay *et al.*, Continued decline of malaria in The Gambia with implications for elimination. *PLoS One.* **5**, e12242 (2010).

18. E. Chanda *et al.*, Impact assessment of malaria vector control using routine surveillance data in Zambia: implications for monitoring and evaluation. *Malar. J.* **11**, 437 (2012).

19. P. Chanda *et al.*, Early results of integrated malaria control and implications for the management of fever in under-five children at a peripheral health facility: a case study of Chongwe rural health centre in Zambia. *Malar. J.* **8**, 49 (2009).

20. L. F. Chaves, M. Hashizume, A. Satake, N. Minakawa, Regime shifts and heterogeneous trends in malaria time series from Western Kenya Highlands. *Parasitology* **139**, 14 (2012).

21. E. Chizema-Kawesha *et al.*, Scaling up malaria control in Zambia: progress and impact 2005-2008. *Am. J. Trop. Med. Hyg.* **83**, 480 (2010).

22. T. D. Clark *et al.*, Incidence of malaria and efficacy of combination antimalarial therapies over 4 years in an urban cohort of Ugandan children. *PLoS One.* **5**, e11759 (2010).

23. J. Cook *et al.*, Serological markers suggest heterogeneity of effectiveness of malaria control interventions on Bioko Island, equatorial Guinea. *PLoS One.* **6**, e25137 (2011).

24. M. H. Craig, I. Kleinschmidt, D. Le Sueur, B. L. Sharp, Exploring 30 years of malaria case data in KwaZulu-Natal, South Africa: part II. The impact of non-climatic factors. *Trop. Med. Int. Health* **9**, 1258 (2004).

25. M. H. Craig, I. Kleinschmidt, J. B. Nawn, D. Le Sueur, B. L. Sharp, Exploring 30 years of malaria case data in KwaZulu-Natal, South Africa: part I. The impact of climatic factors. *Trop. Med. Int. Health* **9**, 1247 (2004).

26. P. De Beaudrap *et al.*, Heterogeneous decrease in malaria prevalence in children over a six-year period in south-western Uganda. *Malar. J.* **10**, 132 (2011).

27. D. B. Dery *et al.*, Patterns and seasonality of malaria transmission in the forest-savannah transitional zones of Ghana. *Malar. J.* **9**, 314 (2010).

28. T. P. Eisele *et al.*, Malaria infection and anemia prevalence in Zambia's Luangwa District: an area of near-universal insecticide-treated mosquito net coverage. *Am. J. Trop. Med. Hyg.* **84**, 152 (2011).

29. G. Feng, J. A. Simpson, E. Chaluluka, M. E. Molyneux, S. J. Rogerson, Decreasing burden of malaria in pregnancy in Malawian women and its relationship to use of intermittent preventive therapy or bed nets. *PLoS One.* **5**, e12012 (2010).

30. L. Gadiaga *et al.*, Conditions of malaria transmission in Dakar from 2007 to 2010. *Malar. J.* **10**, 312 (2011).

31. C. Geiger *et al.*, Declining malaria parasite prevalence and trends of asymptomatic parasitaemia in a seasonal transmission setting in north-western Burkina Faso between 2000 and 2009--2012. *Malar. J.* **12**, 27 (2013).

32. A. A. Gerritsen, P. Kruger, M. F. van der Loeff, M. P. Grobusch, Malaria incidence in Limpopo Province, South Africa, 1998-2007. *Malar. J.* **7**, 162 (2008).

33. A. Gomez-Elipe, A. Otero, M. van Herp, A. Aguirre-Jaime, Forecasting malaria incidence based on monthly case reports and environmental factors in Karuzi, Burundi, 1997-2003. *Malar. J.* **6**, 129 (2007).

34. P. M. Graves *et al.*, Effectiveness of malaria control during changing climate conditions in Eritrea, 1998-2003. *Trop. Med. Int. Health* **13**, 218 (2008).

35. C. Guinovart *et al.*, Malaria in rural Mozambique. Part I: children attending the outpatient clinic. *Malar. J.* **7**, 36 (2008).

36. M. J. Hamel *et al.*, A reversal in reductions of child mortality in western Kenya, 2003-2009. *Am. J. Trop. Med. Hyg.* **85**, 597 (2011).

37. S. D. Hamusse, T. T. Balcha, T. Belachew, The impact of indoor residual spraying on malaria incidence in East Shoa Zone, Ethiopia. *Glob. Health Action* **5**, 11619 (2012).

38. S. I. Hay *et al.*, Clinical epidemiology of malaria in the highlands of western Kenya. *Emerg. Infect. Dis.* **8**, 543 (2002).

39. Y. E. Himeidan, E. E. Hamid, L. Thalib, M. I. Elbashir, I. Adam, Climatic variables and transmission of falciparum malaria in New Halfa, eastern Sudan. *East. Mediterr. Health J.* **13**, 17 (2007).

40. C. C. John *et al.*, Possible interruption of malaria transmission, highland Kenya, 2007-2008. *Emerg. Infect. Dis.* **15**, 1917 (2009).

41. J. F. Jusot, O. Alto, Short term effect of rainfall on suspected malaria episodes at Magaria, Niger: a time series study. *Trans. R. Soc. Trop. Med. Hyg.* **105**, 637 (2011).

42. J. Keating, J. M. Miller, A. Bennett, H. B. Moonga, T. P. Eisele, *Plasmodium falciparum* parasite infection prevalence from a household survey in Zambia using microscopy and a rapid diagnostic test: implications for monitoring and evaluation. *Acta Trop.* **112**, 277 (2009).

43. E. Khosa, L. R. Kuonza, P. Kruger, E. Maimela, Towards the elimination of malaria in South Africa: a review of surveillance data in Mutale Municipality, Limpopo Province, 2005 to 2010. *Malar. J.* **12**, 7 (2013).

44. H. K. Kimbi, D. Nformi, A. M. Patchong, K. J. Ndamukong, Influence of urbanisation on asymptomatic malaria in school children in Molyko, South West Cameroon. *East Afr. Med. J.* **83**, 602 (2006).

45. I. Kleinschmidt *et al.*, Marked increase in child survival after four years of intensive malaria control. *Am. J. Trop. Med. Hyg.* **80**, 882 (2009).

46. B. G. Koudou *et al.*, Effect of agricultural activities on prevalence rates, and clinical and presumptive malaria episodes in central Cote d'Ivoire. *Acta Trop.* **111**, 268 (2009).

47. M. Kristan *et al.*, Variations in entomological indices in relation to weather patterns and malaria incidence in East African highlands: implications for epidemic prevention and control. *Malar. J.* **7**, 231 (2008).

48. H. Lemma *et al.*, Deploying artemether-lumefantrine with rapid testing in Ethiopian communities: impact on malaria morbidity, mortality and healthcare resources. *Trop. Med. Int. Health* **15**, 241 (2010).

49. J. R. Mabiala-Babela, C. Samba-Louaka, A. Mouko, P. Senga, [Morbidity in a pediatric department (University Hospital of Brazzaville): 12 years later (1989-2001)]. *Arch. Pediatr.* **10**, 650 (2003).

50. B. T. Maegga, J. Cox, K. D. Malley, Malaria in the southern highlands of Tanzania: a review of hospital records. *Tanzan. Health Res. Bull.* **7**, 125 (2005).

51. R. Maharaj, D. J. Mthembu, B. L. Sharp, Impact of DDT re-introduction on malaria transmission in KwaZulu-Natal. *S. Afr. Med. J.* **95**, 871 (2005).

52. S. Majambere *et al.*, Is mosquito larval source management appropriate for reducing malaria in areas of extensive flooding in The Gambia? A cross-over intervention trial. *Am. J. Trop. Med. Hyg.* **82**, 176 (2010).

53. J. Manuel Ramos, F. Reyes, A. Tesfamariam, Change in epidemiology of malaria infections in a rural area in Ethiopia. *J. Travel Med.* **12**, 155 (2005).

54. D. P. Mawili-Mboumba *et al.*, Increase in malaria prevalence and age of at risk population in different areas of Gabon. *Malar. J.* **12**, 3 (2013).

55. S. E. Mirghani *et al.*, The spatial-temporal clustering of *Plasmodium falciparum* infection over eleven years in Gezira State, The Sudan. *Malar. J.* **9**, 172 (2010).

56. B. P. Mmbando *et al.*, A progressive declining in the burden of malaria in north-eastern Tanzania. *Malar. J.* **9**, 216 (2010).

57. G. Mtove *et al.*, Decreasing incidence of severe malaria and community-acquired bacteraemia among hospitalized children in Muheza, north-eastern Tanzania, 2006-2010. *Malar. J.* **10**, 320 (2011).

58. J. Mufunda *et al.*, Roll back malaria--an African success story in Eritrea. *S. Afr. Med. J.* **97**, 46 (2007).

59. A. Munier *et al.*, Evolution of malaria mortality and morbidity after the emergence of chloroquine resistance in Niakhar, Senegal. *Malar. J.* **8**, 270 (2009).

60. F. M. Mutuku *et al.*, Impact of insecticide-treated bed nets on malaria transmission indices on the south coast of Kenya. *Malar. J.* **10**, 356 (2011).

61. R. Ndyomugyenyi, P. Magnussen, Trends in malaria-attributable morbidity and mortality among young children admitted to Ugandan hospitals, for the period 1990-2001. *Ann. Trop. Med. Parasitol.* **98**, 315 (2004).

62. L. Ngomane, C. de Jager, Changes in malaria morbidity and mortality in Mpumalanga Province, South Africa (2001-2009): a retrospective study. *Malar. J.* **11**, 19 (2012).

63. P. M. Nyarango *et al.*, A steep decline of malaria morbidity and mortality trends in Eritrea between 2000 and 2004: the effect of combination of control methods. *Malar. J.* **5**, 33 (2006).

64. B. A. Okech *et al.*, Use of integrated malaria management reduces malaria in Kenya. *PLoS One.* **3**, e4050 (2008).

65. E. A. Okiro *et al.*, Malaria paediatric hospitalization between 1999 and 2008 across Kenya. *BMC Med.* **7**, 75 (2009).

66. E. A. Okiro, V. A. Alegana, A. M. Noor, R. W. Snow, Changing malaria intervention coverage, transmission and hospitalization in Kenya. *Malar. J.* **9**, 285 (2010).

67. E. A. Okiro *et al.*, Increasing malaria hospital admissions in Uganda between 1999 and 2009. *BMC Med.* **9**, 37 (2011).

68. E. A. Okiro *et al.*, The decline in paediatric malaria admissions on the coast of Kenya. *Malar. J.* **6**, 151 (2007).

69. L. Ollivier *et al.*, Malaria in the Republic of Djibouti, 1998-2009. *Am. J. Trop. Med. Hyg.* **85**, 554 (2011).

70. W. P. O'Meara *et al.*, Effect of a fall in malaria transmission on morbidity and mortality in Kilifi, Kenya. *Lancet* **372**, 1555 (2008).

71. A. E. Orimadegun, O. Fawole, J. O. Okereke, F. O. Akinbami, O. Sodeinde, Increasing burden of childhood severe malaria in a Nigerian tertiary hospital: implication for control. *J. Trop. Pediatr.* **53**, 185 (2007).

72. M. Otten *et al.*, Initial evidence of reduction of malaria cases and deaths in Rwanda and Ethiopia due to rapid scale-up of malaria prevention and treatment. *Malar. J.* **8**, 14 (2009).

73. N. Protopopoff *et al.*, Spatial targeted vector control is able to reduce malaria prevalence in the highlands of Burundi. *Am. J. Trop. Med. Hyg.* **79**, 12 (2008).

74. A. Roca-Feltrer *et al.*, Lack of decline in childhood malaria, Malawi, 2001-2010. *Emerg. Infect. Dis.* **18**, 272 (2012).

75. A. Rose-Wood, S. Doumbia, B. Traore, M. C. Castro, Trends in malaria morbidity among health care-seeking children under age five in Mopti and Sevare, Mali between 1998 and 2006. *Malar. J.* **9**, 319 (2010).

76. A. K. Rowe, F. Onikpo, M. Lama, D. M. Osterholt, M. S. Deming, Impact of a malaria-control project in Benin that included the integrated management of childhood illness strategy. *Am. J. Public Health* **101**, 2333 (2011).

77. T. L. Russell *et al.*, Impact of promoting longer-lasting insecticide treatment of bed nets upon malaria transmission in a rural Tanzanian setting with pre-existing high coverage of untreated nets. *Malar. J.* **9**, 187 (2010).

78. S. Sarrassat, P. Senghor, J. Y. Le Hesran, Trends in malaria morbidity following the introduction of artesunate plus amodiaquine combination in M'lomp village dispensary, south-western Senegal. *Malar. J.* **7**, 215 (2008).

79. B. L. Sharp *et al.*, Seven years of regional malaria control collaboration--Mozambique, South Africa, and Swaziland. *Am. J. Trop. Med. Hyg.* **76**, 42 (2007).

80. D. I. Stern *et al.*, Temperature and malaria trends in highland East Africa. *PLoS One.* **6**, e24524 (2011).

81. C. G. Sutcliffe *et al.*, Changing individual-level risk factors for malaria with declining transmission in southern Zambia: a cross-sectional study. *Malar. J.* **10**, 324 (2011).

82. H. D. Teklehaimanot, A. Teklehaimanot, A. Kiszewski, H. S. Rampao, J. D. Sachs, Malaria in Sao Tome and principe: on the brink of elimination after three years of effective antimalarial measures. *Am. J. Trop. Med. Hyg.* **80**, 133 (2009).

83. E. A. Temu, M. Coleman, A. P. Abilio, I. Kleinschmidt, High prevalence of malaria in Zambezia, Mozambique: the protective effect of IRS versus increased risks due to pig-keeping and house construction. *PLoS One.* **7**, e31409 (2012).

84. J. Thuilliez, Fever, malaria and primary repetition rates amongst school children in Mali: combining demographic and health surveys (DHS) with spatial malariological measures. *Soc. Sci. Med.* **71**, 314 (2010).

85. L. F. Tseng *et al.*, Rapid control of malaria by means of indoor residual spraying of alphacypermethrin in the Democratic Republic of Sao Tome and Principe. *Am. J. Trop. Med. Hyg.* **78**, 248 (2008).

86. Weekly malaria surveillance in Zimbabwe. *Wkly. Epidemio.l Rec.* **78**, 398 (2003).

87. A. K. Yeshiwondim, S. Gopal, A. T. Hailemariam, D. O. Dengela, H. P. Patel, Spatial analysis of malaria incidence at the village level in areas with unstable transmission in Ethiopia. *Int. J. Health Geogr.* **8**, 5 (2009).

88. G. Zhou *et al.*, Changing patterns of malaria epidemiology between 2002 and 2010 in Western Kenya: the fall and rise of malaria. *PLoS One.* **6**, e20318 (2011).

89. G. Zhou, A. K. Githeko, N. Minakawa, G. Yan, Community-wide benefits of targeted indoor residual spray for malaria control in the western Kenya highland. *Malar. J.* **9**, 67 (2010).
